# Differential expression of host oncogenes in human papillomavirus‐associated nasopharyngeal and cervical epithelial cancers

**DOI:** 10.1002/kjm2.12880

**Published:** 2024-07-29

**Authors:** Santa Sheila, Brown Charles Adoquaye, Akakpo Patrick Kafui, Edusei Lawrence, Hooper Andrew Richard, Quaye Osbourne, Tagoe Emmanuel Ayitey

**Affiliations:** ^1^ Department of Biochemistry, Cell & Molecular Biology/West African Centre for Cell Biology of Infectious Pathogens (WACCBIP) University of Ghana Accra Ghana; ^2^ Department of Medical Laboratory Sciences University of Ghana Accra Ghana; ^3^ Department of Pathology, School of Medical Sciences University of Cape Coast Cape Coast Ghana; ^4^ Pathologists Without Borders Accra Ghana; ^5^ Department of Pathology University of Ghana Medical School, University of Ghana Accra Ghana; ^6^ Korle Bu Teaching Hospital Accra Ghana

**Keywords:** AKT, cervical cancer, IQGAP1, MMP16, nasopharyngeal cancer

## Abstract

Human papillomavirus (HPV)‐related cervical and nasopharyngeal cancers differ in molecular mechanisms underlying the oncogenic processes. The disparity may be attributed to differential expression of oncoproteins. The current study investigated the host oncogenes expression pattern in HPV‐associated cervical and nasopharyngeal cancer. Formalin‐fixed paraffin‐embedded tissues originating from the nasopharyngeal and cervical regions were screened using Hematoxylin and Eosin staining. Genomic DNA and total RNA were extracted from confirmed cancer biopsies and non‐cancer tissues (NC). HPV was detected by PCR using MY09/GP5+/6+ primers. Protein expression levels of AKT, IQGAP1, and MMP16 in HPV‐infected cancers and controls were determined by immunohistochemistry. RT‐qPCR was used to profile mRNAs of the oncogenes. AKT and IQGAP1 proteins were highly expressed in the epithelial cancers compared with the non‐cancer tissues (*p* < 0.05). IQGAP1 and MMP16 mRNAs level was significantly higher in the cancers than in the NC (*p* < 0.05), but not AKT mRNA levels. MMP16 protein was ubiquitously expressed in all tissues. AKT mRNA level was significantly elevated in CC compared with NPC (*p* < 0.001). However, the difference in AKT, IQGAP1 and MMP16 proteins level between CC and NPC was not significant (*p* > 0.05). The oncoproteins expression level between the HPV‐positive and HPV‐negative cancer biopsies showed no significant difference (*p* < 0.05). Current study reports AKT but not IQGAP1 and MMP16 mRNAs differentially expression in cervical and nasopharyngeal cancers, independent of HPV infection status.

## 1 INTRODUCTION

Cancer is one of the leading causes of death globally, and cervical and nasopharyngeal cancers are two of the commonest pathogen‐associated cancers.^1^, ^2^ Human papillomavirus (HPV) infection has been implicated in almost all cervical cancers, however, about 5% of cervical cancers are HPV‐independent.^3^ Based on this, the 2020 WHO Classification of Female Genital Tumors subdivided cervical carcinomas into HPV‐associated and HPV‐independent tumors.^4^ HPV infection has been implicated in up to 35.2% of nasopharyngeal cancers; a subset of head and neck squamous cell cancers (HNCSC) with reported HPV prevalence of up to 68.7% (oral, oropharynx, hypopharynx, tonsillar).^5^ The shift from carcinogen‐induced to HPV‐associated nasopharyngeal cancer in many Western countries has prompted the recognition of 2 distinct clinical types; HPV‐related and HPV‐unrelated HNSCC, with the HPV‐related nasopharyngeal cancer steadily on the rise.^6^

Irrespective of the type of cervical or nasopharyngeal cancer present, disruption of signaling pathways is associated with the pathogenesis of these cancers, and the phosphoinositide‐3‐kinase/ protein kinase B (PI3K/AKT) pathway is one of the major survival pathways of cells implicated in several cancers.^7^ The PI3K/AKT signaling pathway has been reported to play a central role in the virus/host cell crosstalk of HPV‐positive cancer cells.^8^ AKT is a serine/threonine kinase that controls a range of downstream targets involved in cell proliferation, cell growth, cell mobilization, angiogenesis, and cell survival.^9^ Available literature supports the role of HPV E6 and E7 in activating the PI3K pathway through various mechanisms. E6 has been reported to inactivate Phosphatase and tensin homolog (PTEN), leading to increased phosphorylated AKT (pAKT) as well as increased cell proliferation, while E7 inactivates retinoblastoma protein (pRb) leading to increase AKT activity.^10^

The major scaffold protein involved in coordinating and regulating the activities of components of the PI3K‐AKT pathways is the multi‐domain protein, isoleucine glutamine motif containing GTPase‐activating protein 1 (IQGAP1).^11^ Upon receptor activation, IQGAP1 assembles PI4P, PI(4,5)P2, and PI(3,4,5)P3 generating enzymes PI4KIII, PIPKI and PI3K,^12^ with the subsequent generation of PI(3,4,5)P3 lipid messenger 4, which further recruits PDK1 and AKT onto the IQ3 motif of IQGAP1, thus activating AKT to start the downstream signaling.^13^ Aside the PI3K pathway, IQGAP1 also assembles components of the Ras–ERK pathway, and the level of IQGAP1 expression has been shown to play a major role in determining which of the two pathways it scaffolds.^12^ Both knockdown and overexpression of IQGAP1 were shown to diminish ERK activation whereas overexpression of IQGAP1 was reported to enhances AKT activation.^14^ Activated AKT has been reported to cause cancer cell invasion, by increasing cell motility and matrix metalloproteinase (MMP) production.^15^

Available literature supports the relationship between MMP and the invasive ability of malignant tumor.^16^ The high expression of MMP16, a new member of the MMP family, was associated with increased cell proliferation, invasion and poor prognosis in gastric and colorectal cancer patients.^17^ MMP16 is a membrane type‐matrix metalloproteinase (MT‐MMP) that is anchored to the cell membrane with its transmembrane domain located in the cytosol.^18^ MMP16 has been reported to activate MMP2, an important cancer‐associated MMP involved in the invasion and metastasis of a wide range of carcinomas.^19^ However, the role of MMP16 in cervical and nasopharyngeal cancers has not been well elucidated.

Generally, HPV‐related and HPV‐unrelated cervical and nasopharyngeal cancers are known to differ with respect to the molecular mechanisms underlying their oncogenic processes. HPV‐related cancers were reported to show a better response to treatment compared with HPV‐unrelated cancers.^6^ HPV‐positive nasopharyngeal cancers have also been reported to respond better to treatment than HPV‐positive cervical cancers.^20^ Thus, examining the expression patterns of AKT, IQGAP1 and MMP16 mRNA and proteins in HPV‐related cervical and nasopharyngeal cancers will shed more light on HPV‐associated carcinogenesis. Against this background, we aimed to determine the expression pattern of AKT, IQGAP1 and MMP16, in HPV‐associated cervical and nasopharyngeal cancer tissues.

## 2 MATERIALS AND METHODS

### 2.1 Study design and samples

The study was a retrospective cross‐sectional design aimed to determine the expression pattern of AKT, IQGAP1 and MMP16, in HPV‐associated cervical and nasopharyngeal cancer tissues. Four‐hundred and three (403) formalin fixed paraffin embedded (FFPE) cervical and nasopharyngeal biopsies obtained within the period 2019–2021 were screened for the study. The biopsies were screened by a Board‐Certified Pathologist to confirm the presence or absence of cancer using Hematoxylin and Eosin‐stained slides prepared from the FFPE blocks, following a protocol as previously reported. The pathological investigation was carried out at a diagnostic center, Pathology Without Boarders, Accra, Ghana. The confirmed cervical and nasopharyngeal cancer biopsies, and non‐cancer adjacent normal tissues as control were selected and further screened for HPV infection as previously described.^21^

### 2.2 Immunohistochemistry (IHC) and scoring

Immunohistochemical staining was carried out on the Ventana BenchMark GX automated slide‐staining system (Roche, USA) according to the manufacturers protocol using Ventana IHC reagents. Briefly, FFPE tissues sections of 5 μm thickness were baked overnight at 50°C and deparaffinized using EZ prep solution at 75°C for 8 min followed by heat induced epitope retrieval (HIER) in Tris‐EDTA buffer, pH 7.8 at 95°C for 44 min. The sections were incubated separately with 100 µL of 1:200 anti‐AKT, anti‐IQGAP1 and anti‐MMP16 antibodies (Abcam, Cambridge, UK) for 60 min. Negative controls were obtained by performing all IHC steps without the primary antibody.

The sections were further incubated with horseradish peroxidase‐conjugated secondary antibody for the corresponding species of primary antibody. 3,3′‐Diaminobenzidine (DAB) color developing solutions were added to the sections, followed by counterstaining with Hematoxylin for 8 min and bluing reagent for 8 min. The sections were subsequently washed in warm tap water with detergent and dehydrate in graded ethanol and xylene. Tissue sections on the slides were flooded with permanent mounting media and covered with coverslip, and staining intensities evaluated by a Board‐Certified Pathologist. Immunostaining scores were defined as the cell staining intensity (nil = 0; weak = 1; moderate = 2; strong = 3) multiplied by the percentage of labeled cells (0%–100%), producing scores between 0 and 300. A score greater than the mean was defined as “high” immunostaining, whereas a score less than or equal to the mean was categorized as “low” immunostaining, respectively.

### 2.3 Expression pattern of oncogenes using RT‐qPCR

RT‐qPCR amplification was carried out in the QuantStudio 3 system (Thermo Fischer Scientific, Waltham, USA), according to the dye method to determine the mRNA expression patterns of AKT, IQGAP1 and MMP16. For each of the genes, 2 μL of total RNA was added to the master mix containing; OneTaq One‐Step reaction mix, OneTaq One‐Step enzyme mix (25X) (OneTaq® One‐Step RT‐PCR Kit; New England Biolabs, USA), and the respective primers (AKT: F‐5′‐TCT ATG GCG CTG AGA TTG TG‐3′, R‐5′‐CTT AAT GTG CCC GTC CTT GT‐3′; IQGAP1‐F‐5′‐TCCAATAAGATGTTTCTGGGAGAT‐3′, R‐5′‐GATGATTTCACCAATGGAAATGTA‐3′; MMP16‐F‐5′‐AGCACTGGAAGACGGTTGG‐3′, R‐5′‐CTCCGTTCCGCAGACTGTA‐3′) in a total volume of 10 μL. The following amplification conditions were used; 55°C for 10 min, 95°C for 1 min, 95°C for 15 s, 60°C for 1 min, 68°C for 1 min, and 68°C for 5 min according to the protocol of OneTaq® One‐Step RT‐qPCR Kit (BioLabs Inc., Lpswich, New England). All reagent reconstitutions were carried out on ice. GAPDH was used as an internal control and the master mix without template RNA served as a negative control.

### 2.4 Statistical analyses

The GraphPad Prism 9 was used for the statistical analysis. Oncoproteins expression levels were presented as proportion and compared using Fisher's exact test. The fold change in AKT, IQGAP1 and MMP16 expression was calculated using the 2^−ΔΔ*ct*^ and Student's *t*‐test was used to compare the mean difference. Statistical significance was set at *p* < 0.05.

## 3 RESULTS

### 3.1 Immunohistochemical analysis of oncoproteins in HPV‐related cervical and nasopharyngeal cancers

**FIGURE 1:**
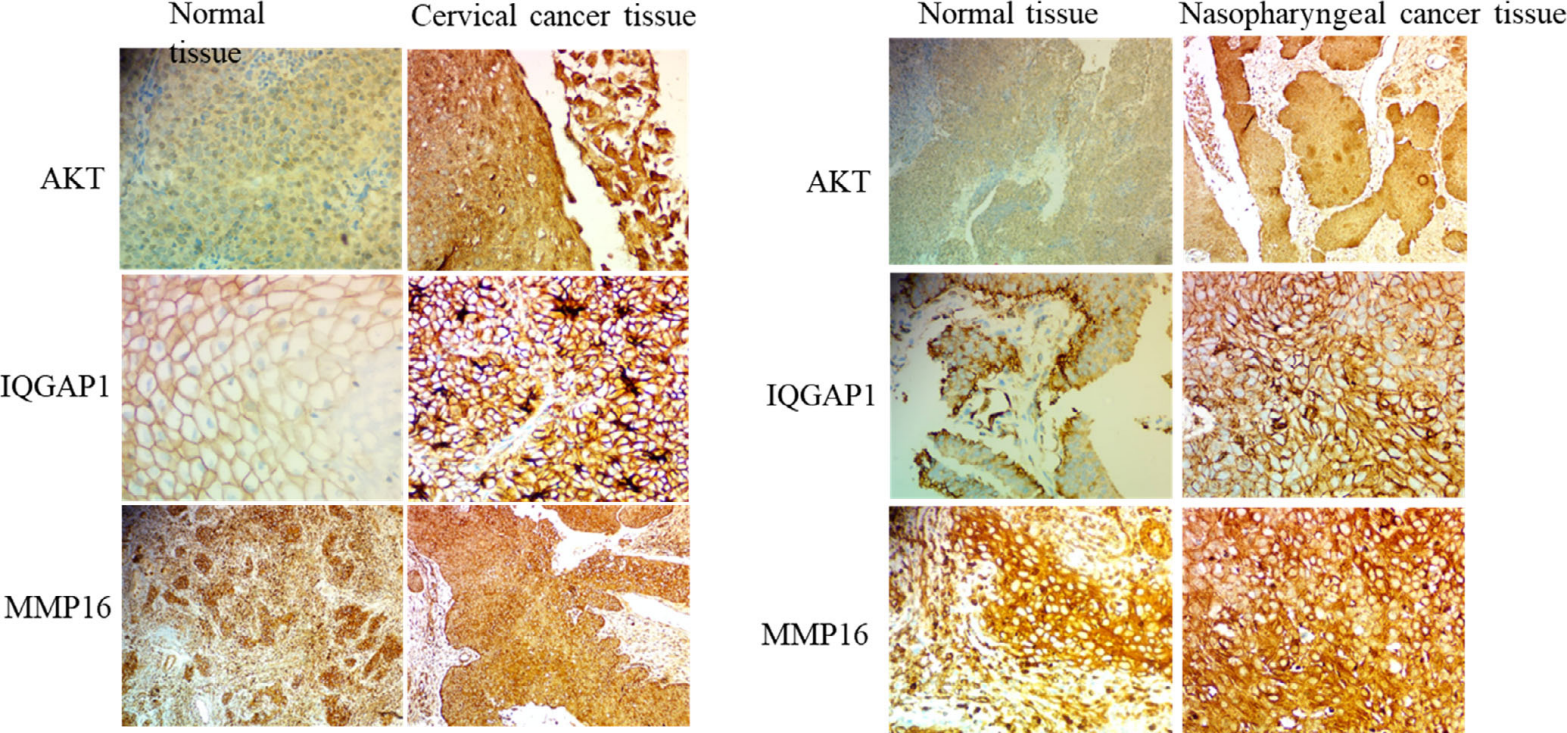
Immunostaining images of cervical and nasopharyngeal cancer tissues showing high and low expression of AKT, MMP16 and IQGAP1. Immunostaining scores were defined as the cell staining intensity (nil, 0; weak, 1; moderate, 2; strong, 3) multiplied by the percentage of labeled cells (0%–100%). This produced a scores between 0 and 300. A score greater than the mean (150) was defined as “high” immunostaining, whereas a score less than or equal to the mean was categorized as “low” expression. Normal tissue with high expression. Cervical cancer tissues magnifications: AKT ×400, MMP16 ×100, IQGAP1 ×400; Cervical normal tissues magnifications: AKT ×400, IQGAP1 ×400, MMP16 ×100. Nasopharyngeal cancer tissues magnifications: AKT ×100, MMP16 ×400, IQGAP1 ×400; Nasopharyngeal normal tissues magnifications: AKT ×100, MMP16 ×400, IQGAP1 ×400.

The positive expression of AKT, IQGAP1, and MMP16 in the epithelial cancers and the normal tissues was identified in the cytoplasm by light yellow, brown‐yellow, or brown coloration (Figure 1). Expression of oncoproteins in the HPV positive epithelial cancer tissues was not different from that of the HPV negative cancer tissues (Table 1). Predominantly, IQGAP1 was highly expressed in both epithelial cancer tissues, whereas MMP16 was ubiquitously expressed in both cancers and normal tissues. In the cancer tissues however, AKT was highly expressed in only 50% (5/10) (Table 2). A comparison of the oncoproteins expression in the cervical and nasopharyngeal cancers showed no significant difference (Table 3).

**TABLE 1:** HPV status and oncoproteins expression in epithelial cancer FFPE biopsies.

Protein	HPV status	No. of tissues	High expression *n* (%)	Low expression *n* (%)	*p*‐Value
AKT	Positive	20	10 (50.0)	10 (50.0)	0.395
	Negative	11	6 (57.0)	5 (43.0)	
IQGAP1	Positive	20	20 (100)	0 (0.0)	1.000
	Negative	11	11 (100)	0 (0.0)	
MMP16	Positive	20	20 (100)	0 (0.0)	1.000
	Negative	11	11 (100)	0 (0.0)	

**TABLE 2:** Expression of oncoproteins in epithelial cancers and normal tissues.

Cancer type	No. of tissues	AKT, *n* (%)		IQGAP1, *n* (%)		MMP16, *n* (%)	
		High	Low	High	Low	High	Low
CC and NPC	20	10 (50.0)	10 (50.0)	20 (100)	0 (0.0)	20 (100)	0 (0.0)
Normal	20	0 (0.0)	20 (100)	0 (0.0)	20 (100)	20 (100)	0 (0.0)
*p*‐value		<0.001		<0.001		1.000	

Abbreviations: CC, cervical; NPC, nasopharyngeal.

**TABLE 3:** Expression of oncoproteins in cervical and nasopharyngeal cancer biopsies.

Sample type	No. of tissues	AKT *n* (%)		IQGAP1 *n* (%)		MMP16 *n* (%)	
		High	Low	High	Low	High	Low
Cervical	10	5 (50.0)	5 (50.0)	10 (100)	0 (0.0)	10 (100)	0 (0.0)
Nasopharyngeal	10	5 (50.0)	5 (50.0)	10 (100)	0 (0.0)	10 (100)	0 (0.0)
*p*‐value		1.000		1.000		1.000	

### 3.2 mRNA expression pattern of AKT, IQGAP1 and MMP16 in cervical and nasopharyngeal cancers and normal tissues

**FIGURE 2:**
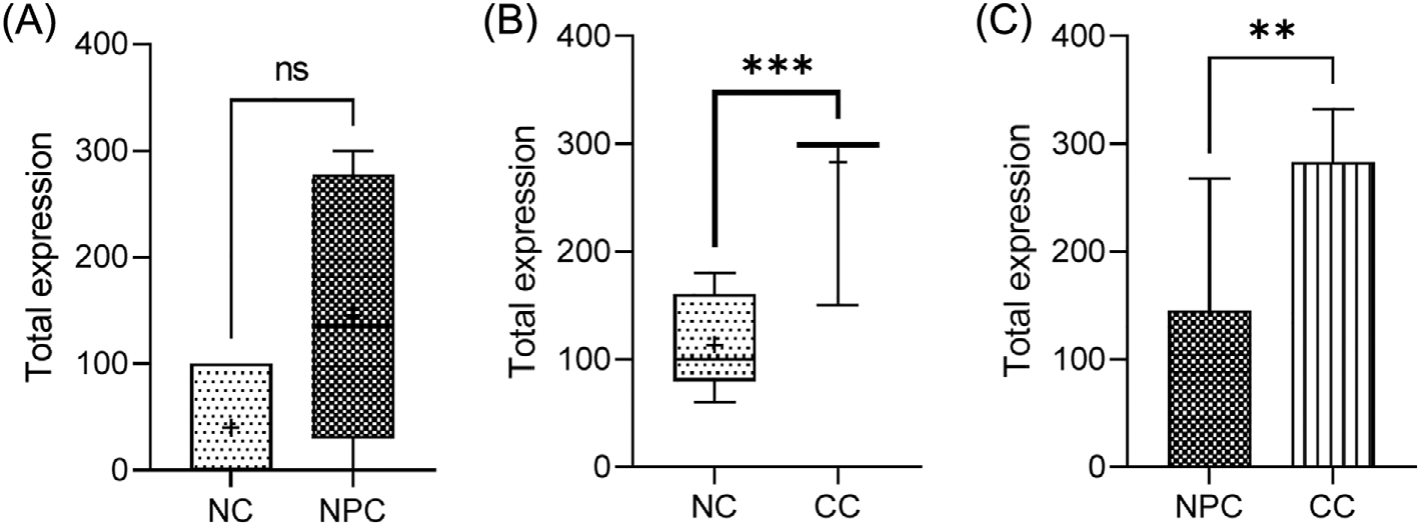
AKT expression in cervical and nasopharyngeal cancers and non‐cancer tissues. CC, cervical cancer; NC, negative control; NPC, nasopharyngeal cancer. **p < 0.01; ***p < 0.001.

The results showed a significantly higher expression level of AKT mRNA in the cervical cancer tissues than the corresponding non‐normal tissues (*p* < 0.001) (Figure 2). In the nasopharyngeal cancer tissues, difference in AKT mRNA level in the nasopharyngeal cancer compared to the normal tissues was not statistically (*p* > 0.05). Nevertheless, AKT mRNA was highly expressed in the cervical than the nasopharyngeal cancer tissues (*p* < 0.01).

**FIGURE 3:**
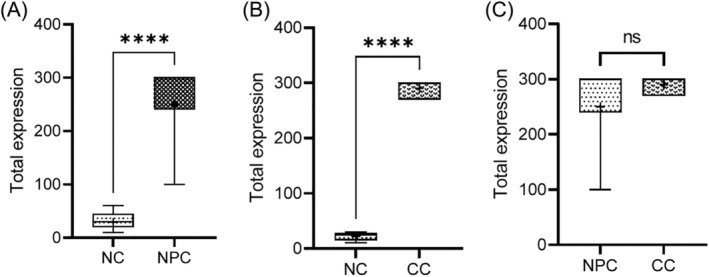
IQGAP1 expression in cervical and nasopharyngeal cancer and non‐cancer tissues. Hundred percent (100%) of the nasopharyngeal and cervical cancer tissues had high expression of IQGAP1, 100% of the nasopharyngeal and cervical non‐cancer tissues had very low expression of IQGAP1. Higher expression of MMP16 was seen in the cervical cancer tissue compared to the nasopharyngeal cancer tissues. Total expression of AKT, IQGAP1, and MMP16 in cervical and nasopharyngeal cancer and non‐cancer tissues. CC, cervical cancer; NC, negative control; NPC, nasopharyngeal cancer.

**FIGURE 4:**
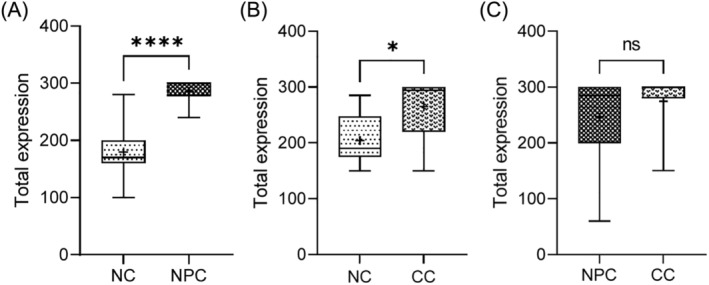
Shows MMP16 expression in cervical and nasopharyngeal cancer and non‐cancer tissues. Hundred percent (100%) of all nasopharyngeal and cervical cancer and non‐cancer tissues had high expression of MMP16. The cancer tissues had higher total expression ranging between 240 and 300 while the non‐cancer tissues had total expression ranging from 150 to 285. Higher expression of MMP16 was seen in the cervical cancer tissue compared to the nasopharyngeal cancer tissues. CC, cervical cancer; NC, negative control; NPC, nasopharyngeal cancer.

The mRNA levels for IQGAP1 in nasopharyngeal and cervical cancer tissues were significantly higher than the corresponding normal adjacent tissues (*p* < 0.001). There was however, no significant difference in IQGAP1 expression level between the nasopharyngeal and cervical cancer tissues (*p* > 0.05) (Figure 3). A high expression of MMP16 mRNA was observed in all the tissues, though, the levels were significantly higher in the nasopharyngeal and cervical cancers than the normal tissues (*p* < 0.05). However, no statistically significant difference was observed in the MMP16 mRNA levels when the epithelial cancers were compared (*p* > 0.05) (Figure 4).

## 4 DISCUSSION

HPV infection‐associated nasopharyngeal cancers have been reported to have a better prognosis than HPV‐infection related cervical cancer,^20^ but whether HPV infection is critical for altered expression of oncoproteins, and differences in prognosis of the epithelial cancers is not clear. The current study reports for the first time a comparative analysis of the expression pattern of AKT, IQGAP1, and MMP16 in HPV‐associated nasopharyngeal and cervical cancers. Altered expression of AKT mRNA is reported in cervical and nasopharyngeal cancer biopsies. AKT mRNA and protein levels were elevated in cervical cancer biopsies than the normal tissues. However, the mRNA level was not different when nasopharyngeal cancer biopsies was compared with the normal tissues. Intriguingly, the mRNA level in the cervical cancer was significantly higher than that of the nasopharyngeal cancer biopsies. An increased expression of AKT has been observed in many cancers, including ovarian, lung, and pancreatic cancers.^22^ The proportion of cancer biopsies with high expression of AKT could be attributed to a possible alternative pathway driving the tumorigenesis. The ERK pathway was found to be an alternative pathway to the PI3K pathway where AKT is activated in most cancers.^23^

Pan et al demonstrated that IQGAP1 is required for both ERK and AKT activation, and that IQGAP1‐dependent ERK or AKT activation is mutually exclusive. Hence the two pathways may actively play a role in cervical and nasopharyngeal cancers.^23^ IQGAP1 was overexpressed in both the nasopharyngeal and cervical cancer biopsies than the normal tissues. Nevertheless, mRNA expression levels between the cancers showed no difference. The expression pattern supports available literature that associated high IQGAP1 protein levels with proliferation and metastasis. Higher expression of IQGAP1 was found in hepatocellular carcinoma than adjacent normal tissues, and the expression was related to poor clinical outcomes and postoperative recurrence in patients.^24^ IQGAP1 as scaffold protein plays a crucial role in the maintenance of cytoskeletal architecture through its interaction with actin cytoskeleton with the adherens junction protein, E‐cadherin‐β‐catenin‐α‐catenin complex. The overexpression of IQGAP1 and direct interaction with membrane‐bound E‐cadherin was suggested as an essential cellular process for cancer metastasis.^25^

MMP16 protein was ubiquitously expressed in cervical and nasopharyngeal cancers as well as non‐cancer tissues. However, the cancer tissues had a significantly higher expression of MMP16 mRNA compared to the non‐cancer tissues though no significant difference in expression levels between the two cancers was observed. MMP16, a new member of the MMP family, has been reported to be upregulated in various cancers compared to normal tissue.^26^ The MMPs are also involved in the normal tissue remodeling, wound healing and angiogenesis processes. Increased expression of MMP16 in cervical and nasopharyngeal cancers may not have a prognostic value. The cervix generally is known to have a relatively large area of immature squamous epithelia known as the transformation zone, and MMPs are required for the regular cellular adaptive changes,^27^ and this could explain the high expression of MMP16 in the normal cervical tissue.

We report no association between HPV infection and expression of AKT, IQGAP1 and MMP16 in the epithelial cancers. A similar study reported a low expression of AKT in HPV infection‐associated penile squamous cell carcinoma.^28^ This suggests that the virus may play a role in the initiation of the cancer but does not drive the progression of the cancer; this phenomenon has been described by Kostov et al.,^29^ as the “hit and run.” The main idea of “hit and run” theory in HPV is that after expression of oncoproteins E6 and E7, the development of cancer is already started, and mutations are accumulated over time through carcinogenesis. Consequently, HPV is not necessary anymore for the maintenance and progression of the cancer.^29^

Although, the current study reported that upregulation of the oncogenes is independent of HPV status, several studies have implicated the viral oncoproteins in cancers. HPV's oncogenic potential is driven by the E6 and E7 oncoproteins, with E6 forming a complex with E6‐AP to target p53 for degradation, leading to uncontrolled cell division and mutation accumulation.^30^ Similarly, E6 interacts with PDZ domain‐containing proteins, such as DLG1 and Scribble, which are involved in maintaining cell polarity and proliferation. The disruption of these interactions can contribute to cellular disorganization and cancer progression.^31^ Alternatively, E7 protein binds to the retinoblastoma protein (pRb) and promotes its degradation, leading to uncontrolled cell cycle progression and DNA replication. E7 also disrupts other cell cycle regulators including cyclins and cyclin‐dependent kinase inhibitors (e.g., p21 and p27), thus promoting cell proliferation. Additionally, E7 induces epigenetic changes, such as DNA methylation and histone modification, which suppress tumor suppressor genes and activate oncogenes.^30^

Both E6 and E7 can impair the DNA damage response, further contributing to genomic instability. The inhibition of DNA repair mechanisms allows the accumulation of mutations, promoting oncogenesis. HPV also employs mechanisms to evade the host immune response, contributing to persistent infection and the opportunity for malignant progression.^32^ E6 and E7 are also able to downregulate the expression of major histocompatibility complex (MHC) molecules, reducing the presentation of viral antigens to the immune system and allowing the virus to persist undetected.^33^ It is known that HPV integration into the host genome can lead to chromosomal instability and disruptions in normal gene function. This integration can result in the deregulation of oncogenes and tumor suppressor genes near the integration site.^34^

In the current study, 92% of the cervical and 77% of the nasopharyngeal cancer tissues were HPV positive. The results support the findings that HPV infection is more associated with cervical cancer than nasopharyngeal malignancies.^35^ The mean age of the patients diagnosed with nasopharyngeal and cervical cancers were 52 and 44 years, respectively. This is consistent with literature as the HPV‐related HNSCC was reported to occur at a younger age (45–50 years).^6^ Similarly, the average age of diagnosis of cervical cancer globally was reported to be 53 years ranging from 44 to 68 years with women in lower resource setting mostly having an advanced age of diagnosis.^36^ The relatively younger age of our cervical cancer patients could be due to the high prevalence of nonmarital early sexual initiation (56.9%) among Ghanaian women which increases the risk of HPV infection at a younger age.^37^

Also, a 5‐year comprehensive cervical cancer prevention scheme that was launched to facilitate the early detection and prevention of cervical cancer in Ghana,^38^ could have contributed to the early age of diagnosis. Our nasopharyngeal cases were also found to be overrepresented among males (77%) than females (23%) and the result aligns with findings from a study where nasopharyngeal cancer was found to be prevalent among men and is thought to be as a result of increased tobacco, alcohol, and pickled food consumption, and increased use of benzene and formaldehyde among males.^39^

## 5 CONCLUSION

Prevalence of HPV‐positive cervical cancer was higher than that of nasopharyngeal cancer. Oncogenes, *AKT* but not *IQGAP1* and *MMP16* mRNAs were differentially expressed in the epithelial cancers. However, expression of the mRNAs and oncoproteins were independent of HPV infection status.

## CONFLICT OF INTEREST STATEMENT

The authors declare that they have no competing interests.

## ETHICS STATEMENT

Ethical approval was sought from the Ethics Committee of Basic and Applied Sciences (ECBAS), University of Ghana, with identification number ECBAS 045/21‐22.
